# The Effects of an Incentive Boost on Response Rates, Fieldwork Effort, and Costs across Two Waves of a Panel Study

**DOI:** 10.12758/mda.2020.04

**Published:** 2020

**Authors:** Katherine A. McGonagle

**Affiliations:** University of Michigan, Institute for Social Research

**Keywords:** data collection, incentives, nonresponse, response rate, contact strategies, fieldwork effort, panel study

## Abstract

This paper describes the association between an incentive boost and data collection outcomes across two waves of a long-running panel study. In a recent wave, with the aim of achieving response rate goals, all remaining sample members were offered a substantial incentive increase in the final weeks of data collection, despite uncertainty about potential effects on fieldwork outcomes in the following wave. The analyses examine response rates and the average number of interviewer attempts to complete the interview in the waves during and after the incentive boost, and provide an estimate of the cost of the incentives and fieldwork in the waves during and following the boost. The findings provide suggestive evidence that the use of variable incentive strategies from one wave to the next in the context of an ongoing panel study may be an effective strategy to reduce nonresponse and may yield enduring positive effects on subsequent data collection outcomes.

This paper examines the use of an increase in study incentives near the end of the field period in a recent wave of the Panel Study of Income Dynamics (PSID), a long-running household panel study of U.S. families, and the association with data collection outcomes, including respondent cooperation, fieldwork effort (as assessed by number of interviewer attempts to complete the interview), and fieldwork costs in the following wave.

The beneficial effects of providing incentives in exchange for participation in interviewer-administered surveys are well documented (e.g. see Laurie & Lynn, 2009; Singer & Ye, 2013). Substantial research based on longitudinal studies finds that incentives are associated with higher response rates (e.g., Fumagalli, Laurie, & Lynn, 2010; Hsu, Schmeiser, Haggerty et al., 2017; Martin, Abreu, & Winters, 2001; McGonagle & Freedman, 2017; McGonagle, Couper, & Schoeni, 2011; McGonagle, Schoeni, & Couper, 2013; Rodgers, 2002) and fewer attempts to complete an interview in the wave they are offered (e.g., Markesich & Kovac, 2003; McGonagle et al., 2013).

Despite numerous studies on the effects of incentives, the topic of differential incentive strategies in the context of ongoing panel studies has received little attention (see Singer & Ye, 2013). A handful of studies have found that incentives provided in a study’s first wave have enduring effects on panel retention in subsequent waves (e.g., Goldenberg, McGrath, & Tan, 2009; James, 1997; Lengacher, Sullivan, Couper, et al., 1995; Mack, Huggins, Keathley, et al., 1998; McGrath, 2006; Pforr, Blohm, Blom, et al., 2015; Singer, Van Hoewyk, & Maher, 1998). While these findings indicate that the positive effects of incentives offered at study entry may persist across waves, it is unclear whether this applies to incentives offered later in a panel’s history. In particular, the consequences of providing variable incentive amounts across sample members, or temporarily increasing incentive amounts within a particular wave – on data collection outcomes in future waves – are largely unknown.

During 2015, a differential incentive strategy was implemented in the PSID. As with panel studies across the world (De Leeuw, Hox, & Luiten, 2018), in recent waves PSID has experienced increased difficulty making contact with sample members and gaining their cooperation to complete the interview. In 2015, the study was faced with a substantially higher number of attempts by interviewers to make contact with sample members compared to prior waves, resulting in a high proportion of outstanding sample at risk for nonresponse late in the field period. In the final weeks of data collection, a substantial incentive increase was offered to all remaining sample members. This strategy was undertaken to maintain the study’s high response rate in the current wave, despite uncertainty about the impact on data collection outcomes in the following wave when the incentive was returned (i.e., reduced) to the baseline amount.

This paper examines the overall utility of the incentive boost across two waves of data collection in the PSID. The goal is to contribute to the “urgent need” identified by Laurie and Lynn (2009) to “extend the research knowledge base… to use survey budgets effectively and wisely when choosing respondent incentive strategies for longitudinal surveys.” Using observational panel data, the following questions are considered: Is there evidence that a large incentive boost reduces nonresponse in the wave it is provided? What are the data collection outcomes in the wave following an incentive boost, when the incentive is returned to the baseline amount, including response rate and average number of interviewer attempts to complete the interview, and what percentage of respondents respond to the initial incentive, and what percentage respond only when the incentive is increased? Finally, the cost implications of the incentive boost are examined. What were the relative costs of the increased incentive in the current wave, and did these higher costs endure in the following wave? Limitations for the findings and next steps for research are described.

## Methods

This report draws on production data collected during the 2015 and 2017 waves of the Panel Study of Income Dynamics (PSID). The PSID is a longitudinal study of a nationally representative sample of U.S. families that began in 1968 and collects a variety of data on economic, health, and social behavior (see McGonagle, Schoeni, Sastry et al., 2012 for more information). Interviews have been conducted annually 1968–1997 and biennially since 1999 by professional interviewers employed by the Survey Research Operations group at the Survey Research Center within the Institute for Social Research at the University of Michigan. The study has achieved high wave-to-wave re-interview response rates, exceeding 93% in most waves. Data collection occurs in odd-numbered years between about March 1 and December 31 over the course of 44 weeks. December 31 is a firm end date for the collection of data each wave because the instrument questionnaire content focuses on specific time periods within the current calendar year.

Since 2003, the mode of data collection for approximately 97% of the sample has been computer-assisted telephone interview with in-person visits made to a small fraction of sample members. The study interviews one adult respondent in each family, typically the individual who is most knowledgeable about the family finances (known as the “Reference Person”). Interviewers attempt to contact respondents primarily using telephone (comprising more than three-quarters of all contact attempts in 2015 and 2017), as well as by sending a small number of email and text messages. The average interview length was about 75 minutes in both 2015 and 2017. During 2015 and 2017, interviews were completed with 9,048 and 9,155 families with overall wave-to-wave re-interview response rates (i.e., response rates among those who had participated in the prior wave) of 93% and 94%, respectively.

### Use of incentives.

Since the inception of the study, post-paid monetary incentive payments have been offered to respondents in exchange for the completion of an interview. The incentive payment is typically provided by bank check to the family member who completes the interview. The general strategy in selecting the incentive amount is to offer an amount that roughly aligns with the interview length (i.e., roughly $1 USD for each minute of content) and to maintain a static amount for two waves that is modestly raised every third wave. These increases are intended to adjust for inflation and any increase in the length or general burden of the survey request. Sample members are provided with advance notice of the incentive amount being offered to complete the interview in an informational letter sent prior to the start of each wave of data collection. All subsequent messages sent to sample members requesting their participation reference the incentive. Historically, the incentive offer has remained unchanged throughout a wave of data collection, and all sample members have been offered the same incentive amount. In 2015, a baseline incentive of $70 USD was offered to 8,889 families who also participated in the prior wave (i.e., “re-interview cases”).

During 2015, nearly 15% (1,322 cases) of the 8,889 re-interview cases had not completed their interview with approximately six weeks remaining in the production period. Reflecting the growing difficulty in recent waves of making contact with sample members in telephone studies, by comparison, with the same amount of time remaining in the 2013 wave, a much smaller fraction (6.6%) had not completed their interview. With the goal of achieving the target response rate for the 2015 wave, all remaining cases were offered a large incentive increase from $70 USD to $150 USD. The selection of the amount of the incentive increase was to make the survey request highly salient and reduce perceived barriers to participation by the study’s end date. The incentive boost was communicated to respondents in various ways, including an announcement through a postcard sent via U.S. postal mail, through messages left by interviewers on telephones and cell phones, and through an email and text message. The $150 USD incentive remained in effect throughout the remaining weeks of the field period.

At the start of data collection the following wave (2017), the baseline incentive offer was restored. In this wave, the baseline incentive offer was $75 USD, an increase of $5 USD over the $70 USD baseline incentive offered at the start of 2015, following the convention of modest increases in the baseline incentive every third wave. At the end of the 2017 field period with six weeks remaining, the incentive offer was again increased to $150 USD for all remaining sample members.

## Results

Table 1 presents response rates and field effort in the current and subsequent waves for respondents who were offered the incentive boost in the final six weeks of production during 2015. Field effort is defined as the average number of total attempts by the interviewer using telephone, email and text message required to complete the interview. The first column provides information on the fieldwork outcomes in the 2015 wave (“Current wave”) for the 1,322 cases offered the 2015 incentive boost.

As shown in Table 1, the $150 USD incentive boost in 2015 had a positive impact on study participation with the majority of respondents (59.9%) completing the interview by the end of the field period, allowing response rate goals to be met. The second column provides information on fieldwork outcomes in the 2017 wave (“Next wave”) for the subset of respondents who completed the 2015 interview after being offered the incentive boost. The key question is whether data collection outcomes for those now being offered $75 USD to complete their interview, half as much, were negatively affected. The results show that there is no evidence that respondents were reluctant to participate given the reduced incentive amount. The vast majority of respondents – nearly 89% – who received the $150 USD incentive boost in 2015 continued to participate during the 2017 wave. Moreover, field effort in the 2017 wave actually decreased substantially for those receiving the incentive boost compared to the 2015 wave, dropping from an average number of 82.8 interviewer attempts to complete the interview in 2015 to an average of 33.7 interviewer attempts in 2017.

A second key question is what proportion of respondents who received the incentive boost in 2015 completed the interview in 2017 for the baseline incentive of $75 USD, and what proportion delayed participation until being offered $150 USD. As shown in the table, the vast majority of these respondents – 73.3% – completed their 2017 interview for the baseline incentive offer of $75 USD. Another 26.7% of those who required $150 USD to respond in 2015 responded in 2017 only after again being offered $150 USD near the close of the field period.

Among those completing their interview for the $75 USD baseline incentive, the average number of interviewer attempts was only about 16.0, compared to about 65.0 interviewer attempts on average for those cases who again delayed their participation for the $150 USD at the end of the 2017 field period (not shown in table).

The final question considers the cost-implications of the incentive boost. A concern for survey organizations is that respondents who receive an incentive increase in one wave may resist completing the interview if offered a lower amount in a future wave, leading such increases to be permanent. Did the 2015 incentive boost lead to enduring costs in the following wave? A basic estimate of the fieldwork effort and incentive costs in each wave for the 780 respondents who participated in both waves was generated. A cost-per-interviewer-attempt estimate of $5.50 USD was derived based on the average hourly wage of an interviewer ($22 USD) and the assumptions that interviewers could make four attempts per hour and that each attempt type (telephone, email and text message) required the same amount of time ($22 USD/4 attempts = $5.50 USD). As shown in Table 2, using the average number of interviewer attempts across the 780 cases (i.e., average attempts of 82.8 in 2015 and 33.7 in 2017), fieldwork costs for these respondents are estimated at $355,212 USD in 2015 and at $144,573 USD in 2017. Incentive costs in 2015 were $117,000 USD (i.e., all 780 respondents required $150 USD). In 2017 incentive costs for these 780 respondents dropped by more than one-third to $74,120 USD (i.e., 73.3% responding during the baseline offer of $75 USD and 26.7% responding for the increased offer of $150 USD). Summing costs attributable to fieldwork effort and incentive payments yields total costs of $472,212, or $605 per case in 2015, and $218,693 or $280 per case in 2017, a decline of more than 50% in total costs. In sum, both incentive costs and fieldwork costs decreased substantially for cases receiving the increased incentive in the subsequent wave.

## Discussion

The goals of the current study were to examine the effects of an increased incentive on cooperation late in the field period of a long-running panel study, and trace its effects to response rates and fieldwork outcomes in the following wave. An important limitation to note at the outset is the lack of a randomly selected control group in the assignment of the incentive boost. Since all late-responding sample members were offered an increased incentive, it is not possible to compare outcomes with those who were not offered a higher incentive amount. A second limitation is that the results of the current study are drawn from the experience of a specific ongoing panel study comprising U.S. adults whose families have participated across many decades, making the generalizability of the results to other study designs uncertain.

Despite these limitations, several key findings have emerged from this descriptive analysis. First, the incentive boost was successful in achieving the main operational goal of meeting response rate targets in the wave it was implemented, inducing cooperation from a high percentage of respondents late in the field period. Second, there is no evidence that the increased incentive negatively affected data collection outcomes among respondents offered a lower initial incentive in the subsequent wave, with nearly 89% completing an interview. Moreover, those receiving the incentive boost required substantially less field effort in the following wave to complete their interview than was needed to finalize their interview in the wave they received the boost. Third, contrary to the concern that the costs of the incentive boost would endure in the subsequent wave, costs substantially declined, with the majority of respondents completing the 2017 interview for the baseline offer with about one-third fewer contact attempts than needed in the prior wave.

In providing suggestive evidence that the positive effects of monetary incentives may persist over time, this descriptive analysis is consistent with the handful of studies on this topic in the literature (Jäckle & Lynn, 2008; Mack et al., 1998; Scherpenzeel, Zimmermann, & Budowski, 2002). In the current study, the concern that those who were offered a substantially higher incentive at a point in time would then delay their participation until the same amount was offered was not realized for the majority of respondents.

In the context of a long-running panel study, the offer of a substantial incentive increase may induce survey participation by highlighting to respondents the legitimacy of the study and the value of their participation. Moreover, interviewers likely gain confidence from the raised incentive when making contact with “difficult” respondents who have evaded many prior attempts. Such mechanisms have been suggested to also underlie the beneficial impact of respondent materials, such as letters sent by survey organizations in advance of data collection (De Leeuw, Callegaro, & Hox, 2007). These positive effects may carry-over to subsequent requests for survey participation, potentially by building rapport and good-will, as well as through the elicitation of principles of social exchange and reciprocation (see e.g., Dillman, Smyth, & Christian, 2009).

A note on the choice of the amount of the incentive increase is in order. In the selection of initial monetary incentive amounts and subsequent magnitudes of increases that may occur during fieldwork, survey practitioners have little research evidence on which to draw. This can be traced to the challenges of mounting experiments during active data collection which may have uncertain effects on study goals, as well as the highly contextualized nature of study designs where multiple factors must be considered in the selection of incentive amounts, including respondent characteristics, interview length and burden, and budgetary constraints. Our goal was to implement a highly salient incentive increase in order to reduce respondent barriers to participation and achieve a particular response rate goal by the firm end date of the study. Designing and implementing experimental studies on this topic to better understand the relative effectiveness of different orders of magnitudes of incentive increases would be of high value to the field.

In summary, the findings of this study are consistent with prior research documenting the positive effects of incentives on data collection outcomes. The results additionally provide suggestive evidence that using variable incentive strategies over waves of fieldwork in the context of a large national panel study may be an effective strategy to maximize response rates and yield enduring positive effects on subsequent participation and field effort. An important consideration for ongoing panel studies in future research is how individual characteristics of sample members may affect responsivity to differential incentives and influence sample bias over subsequent waves. Future research should replicate these findings using experimental methods to better understand the mechanisms through which these outcomes occur.

## Figures and Tables

**Table 1:** Fieldwork outcomes over two waves for re-interview respondents offered an incentive boost

	Current wave	Next wave
	2015 (n=1,322)	2017 (n=780)
Response rate^1^	59.9%	88.6%
Number of attempts among respondents (mean)	82.8	33.7
Incentive amount required for response		
$150 (boost)	100.0%	
$75 (baseline offer)		73.3%
$150 (end of study offer)		26.7%
Total	100.0%	100.0%

1 Of the 791 repondents who completed the 2015 interview following the $150 USD incentive boost, 11 were ineligible for the study 2017

**Table 2:** Cost estimates of fieldwork effort by wave

	Current wave	Next wave
Cost parameters	2015	2017
Number of cases responding in both waves	780	
Average cost per interviewer attempt	$5.50	
Total interviewer attempts (mean)	82.8	33.7
Average cost of interviewer attempts	$355,212	$144,573
Average cost of incentive payments	$117,000	$74,120
Total cost	$472,212	$218,693
Cost per case	$605	$280
